# Biodegradable, pH-Sensitive Hollow Mesoporous Organosilica Nanoparticle (HMON) with Controlled Release of Pirfenidone and Ultrasound-Target-Microbubble-Destruction (UTMD) for Pancreatic Cancer Treatment

**DOI:** 10.7150/thno.36135

**Published:** 2019-08-14

**Authors:** Feng Gao, Jianrong Wu, Shiwei Niu, Ting Sun, Fan Li, Yun Bai, Lifang Jin, Lizhou Lin, Qiusheng Shi, Li-Min Zhu, Lianfang Du

**Affiliations:** 1 Department of Ultrasound, Shanghai General Hospital, Shanghai Jiaotong University School of Medicine, Shanghai 201600, P.R. China.; 2 College of Chemistry, Chemical Engineering and Biotechnology, Donghua University, Shanghai 201620, P.R. China.

**Keywords:** Hollow mesoporous organosilica nanoparticles, biodegradability, ultrasound target microbubble destruction, extracellular matrix, gemcitabine gatekeeper

## Abstract

The dense extracellular matrix (ECM) and hypovascular networks were often found in solid pancreatic tumors form an impenetrable barrier, leading to limited uptake of chemotherapeutics and thus undesirable treatment outcomes.

**Methods**: A biodegradable nanoplatform based on hollow mesoporous organosilica nanoparticle (HMON) was designed as an effective delivery system for pirfenidone (PFD) to overcome the challenges in pancreatic tumor treatment. By varying pH producing a mildly acidic environment to emulate tumor cells, results in cleavage of the acetal bond between HMON nanoparticle and gating molecular, gemcitabine (Gem), enabling its controlled release.

**Results**: The *in vitro* and *in vivo* immunocytochemistry evaluations demonstrated an excellent ECM regulation efficacy of the nanoplatform and therefore the improved penetration of drug into the cells. The technique employed was especially enhanced when mediated with ultrasound target microbubble destruction (UTMD). Evaluations culminated with pancreatic cancer bearing mice and demonstrated therapeutic efficacy, good biodegradability, and negligible systemic toxicity.

**Conclusion**: the designed Gem gated biodegradable nanosystem is expected to provide an alternative way of improving antitumor efficacy by down-regulation of ECM levels and offers a passive-targeted therapy for pancreatic cancer treatment.

## Introduction

Desmoplastic stroma is a prominent feature of pancreatic cancer differing from other types of tumors 1-3. Its unique structure results in the formation of a physical barrier, thus preventing the penetration of chemotherapeutic drugs and other formulations 4, 5. In this type of stroma, pancreatic stellate cells (PSCs) are involved in creating an ECM-enriched micro-environment through excreting multiple proteins (*e.g.,* collagen I and fibronectin) 3, 6, 7.Therefore, down-regulation of ECM deposition is of importance as an effort to control the penetration and perfusion of different therapeutic formulations into the tumor microenvironment. Previous studies demonstrated that overcoming desmoplasia *via* regulating PSCs was an effective approach to weaken the physical barrier and therefore improve the penetration of drugs and nano-carriers for chemotherapy 8, 9. Gemcitabine (Gem) has been proved as a first-line clinical drug for pancreatic cancer treatment. Various treatments including Gem alone or the combination of Gem with other chemotherapeutics, such as pirfenidone (PFD), a FDA approved anti-fibrosis drug used for idiopathic pulmonary fibrosis, were adopted for pancreatic cancer treatment. However, vascular deficiency limited the accumulation of drugs in the tumor 10 and thus was ineffective at suppressing tumor metastases. A novel nanomedicine system that not only regulates the pancreatic tumor micro-environment in a tumor stroma, but also enhances the accumulation of chemotherapeutics in the tumor is an attractive form of pancreatic cancer treatment.

As an alternative to traditional porous nanomaterials, a variety of different mesoporous silica nanoparticles (MSNs) and porous hollow silica nanoparticles have been studied as a drug delivery system because of their good *in vitro* and *in vivo* biocompatibility 11-18. Despite tremendous research in developing new silica-based nanosystems for cancer nanotheranostics, unpredictable toxicity (*i.e.,* uncontrolled biodegradability of the silica framework (Si-O-Si), unclear retention times *in vivo*, biosafety, and pathway, *etc.*) significantly limits the potential for clinical transformation 19, 20. It was recently reported that incorporation of disulfide bonds (-S-S-) into the silica framework helped to achieve a fast degradation of organic/inorganic hybrid nanoparticles through intracellular glutathione (GSH) stimulation 21-23. For an instance, Yu *et al.* reported a structure-dependent, GSH-responsive biodegradable, dendritic mesoporous organosilica nanoparticle which performed as an efficient delivery platform for therapeutic biomacromolecules in cancer treatment 24. Shi *et al.* discovered an organic-inorganic hybridized hollow mesoporous organosilica nanoparticle (HMON) based on a “chemical homology” mechanism for guest drug molecule encapsulations 25. Inspired by these work, HMON with a disulfide bonded hybrid framework was selected as a model drug delivery system for PFD in this study.

It is well known that the hypovascular networks allow less perfusion than surrounding normal pancreatic tissues causing ineffectiveness in chemotherapy 10, 26, 27. To address this issue, passive targeting techniques like manipulation-ultrasound-triggered microbubble destruction (UTMD) have been commonly used to disrupt surrounding tissues so as to enhance the penetration 28-32. It has been proved that ultrasound was able to burst those gas filled microbubbles and create micro-steam, which enlarged the capillary gaps and induced transient pores on cell membranes 33, 34. Further, UTMD was shown to help promote the clathrin-based endocytosis of nanoparticles 35.

**Figure 1:**
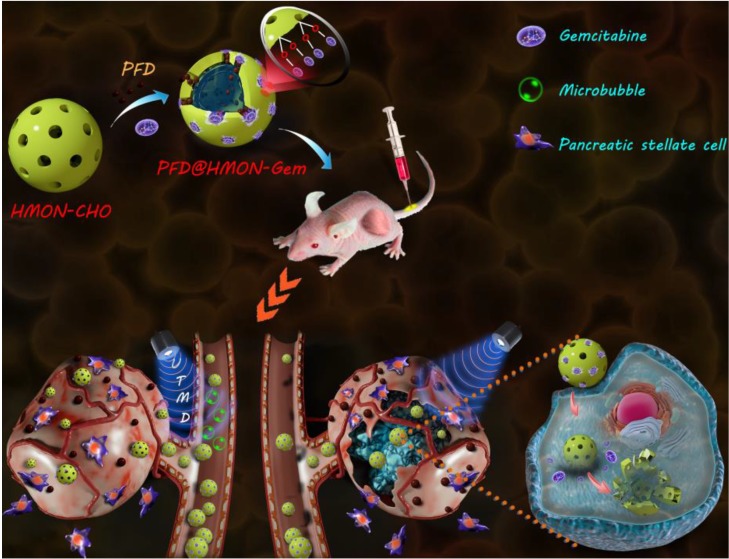
Schematic illustration of a PFD@HMON-Gem nanoplatform for down-regulation of multiple components of tumor ECM and UTMD-mediated chemotherapy for pancreatic cancer therapy.

In this study, we designed a HMON nanosystem to deliver the anti-fibrosis drug (PFD) to down regulate the expression of multiple ECM components for high-performance tumor therapy. HMON nanoparticles with tumor-sensitive biodegradability were selected as ideal drug carriers due to their biologically active disulfide bond framework and large surface area. Gem, a small molecular antitumor drug with active hydroxide groups, was further incorporated onto the pore channel of PFD-loaded HMON nanoparticles *via* a reaction with an aldehyde functional group on the surface. Gem in this design was used as the chemotherapeutics as well as the “gatekeeper.” As a pH-sensitive gatekeeper, the resultant acetal bond on the PFD@HMON-Gem complex can be cleaved in the mild acidic micro-environment of the tumor ECM to adequately release PFD. Effective treatment with PFD will result in down-regulation of the expression of multiple ECM components and eventually enhance the penetration of nanomedicines into the deep tumor tissue (Figure 1). Additionally, the UTMD technique was also employed to further improve the penetration of HMON nanomedicines for better chemotherapeutic activity. It is believed that UTMD in combination with chemotherapy creates a tumor ECM microenvironment-responsive HMON system will serve as a powerful platform for such nanomedicines to be used in pancreatic or other ECM-enriched tumor treatment.

## Materials and Methods

### Chemicals and reagents

Tetraethylorthosilicate (TEOS), ammonia solution (25-28%), p-toluenesulfonic acid (PTSA), anhydrous potassium carbonate, and triethanolamine (TEA) were purchased from Sinopharm Chemical Reagent Co. Cetanecyltrimethyl ammonium chloride (CTAC), 3-aminopropyltriethoxysilane (APTES), N-(3-(dimethylamino)propyl)-N'-ethylcarbodiimide hydrochloride (EDC), N-hydroxysuccinimide (NHS), pirfenidone (PFD), and 4-formylbenzoic acid were obtained from Aladdin Chemistry, Co., Ltd. (Shanghai, China). Gemcitabine (Gem), Rhodamine B (RB), Bis (3-triethoxysilylproyl) disulfide (BTDS), and glutathione (GSH) were provided by Sigma-Aldrich (MO, USA). Dulbecco's modified Eagle's medium (DMEM), fetal bovine serum (FBS), pennicilin, streptomycin, and trypsin were supplied by Gibco Life Technologies (Grand Island, New York, USA). 1,1'-dioctadecyl-3, 3, 3',3'-tetramethyl indotricarbocyanine iodide (DIR) was provided by Biotium (Hayward, CA, USA), 4',6-diamino-2-phenylindole (DAPI) from Beijing Solarbio Science & Technology (Beijing, China). The Annexin V-FITC/PI cell apoptosis analysis kit was purchased from BD Bioscience (New Jersey, New York, USA) and cell counting kit 8 (CCK-8) from Dojindo Molecular Technology (Japan). The terminal deoxynucleotidyl transferase-mediated dUTP nick-end labeling (TUNEL) Kit, hematoxylin & eosin (H&E) and Lyso Tracker red were provided by Beyotime Biotechnology (Shanghai, China) and fluorescein isothiocyanate (FITC) by Thermofisher Scientific (Shanghai, China).

### Synthesis of degradable hollow mesoporous organosilica nanoparticles (HMON)

The disulfide-bridged hollow mesoporous organosilica nanoparticles were prepared as previously reported following the “chemical homology” method 25, 36. In brief, CTAC aqueous solution (10 g, 10 wt.%) was first mixed with TEA aqueous solution (0.5 g, 10 wt.%) under mild magnetic stirring at 95 ºC. TEOS (1 mL) was then added dropwise into the above solution. After stirring for 1 h, a pre-mixed solution of TEOS (0.5 mL) and BTDS (0.6 mL) was added and stirred for another 4 h to form a mesoporous silica/organosilica (MSN@MON) core-shell structure. The product was collected by centrifugation, washed with ethanol and 30 mL of ethanol and water (3:7, v/v) for several times and then extracted with HCl and ethanol (1:10) solution at 60 ºC for 8 h in triplicate to remove excess CTAC. In the following etching process, the obtained MSN@MON mixture in 2 mL of water was re-dispersed into 20 mL of water, followed by the addition of 0.5 mL of ammonia solution (25 wt.%). After reacting for 3 h at 95 ºC, the final HMON nanoparticle product was recovered by centrifugation and washed with water, followed by drying in vacuum for 24 h.

### Synthesis of HMON-CHO

To obtain the aldehyde-functionalized HMON nanoparticles, amine functional groups were first introduced and then reacted with 4-formylbenzoic acid. More specifically, HMON (100 mg) was dispersed in 80 mL of ethanol mixed with 1.2 mL of APTES. The mixture was refluxed at 80 ºC overnight and the product (HMON-NH_2_) was collected and washed with ethanol for 3 times, followed by drying at 60 °C under high vacuum (0.07 psi) overnight. Next, 0.5 g of the obtained HMON-NH_2_ nanoparticles were suspended in 20 mL of a mixture of water/DMSO (4:1, v/v), into which 4-formylbenzoic acid (15.0 mg), EDC (20 mg), and NHS (10 mg) were added under stirring. After 24 h, the final dispersion was dialyzed for 3 d using a cellulose membrane (cut off MW: 3500 Da) to obtain the HMON-CHO nanoparticles.

### PFD loading, Gem capping, and *in vitro* PFD release

For PFD loading, 25 mg of HMON-CHO nanoparticles were dispersed in 50 mL deionized water containing 10 mg of PFD, and stirred for 12 h at room temperature. The PFD loaded HMON-CHO (designated as PFD@HMON-CHO) was obtained by centrifugation and washed with water several times to remove any unreacted PFD. The PFD loading capacity was measured by UV-vis-NIR spectrometry at λ = 263 nm. The PFD@HMON-CHO nanoparticles were further capped with Gem (containing a hydroxide functional group) *via* reaction with aldehyde at pH 7.4. Briefly, 100 mg of PFD@HMON-CHO was dispersed in 100 mL of toluene. Then, 70 mg of Gem with 20 mg of PTSA (as a catalyst) were added, simultaneously. After vigorous stirring for 14 h, the water byproduct was removed by an oil/water separator and toluene was removed *via* a rotavapor. The final product designated as PFD@HMON-Gem was washed with potassium carbonate (1 wt.%, 80 mL) to remove any remaining unreacted PTSA and Gem. Meanwhile, HMON-Gem was synthesized in a similar process.

### Characterization

Transmission electron microscope (TEM) images were obtained using a JEOL JEM-2010 unit at an acceleration voltage of 200 kV (JEOL USA, Inc., Peabody, MA, USA). The Fourier transform infrared (FTIR) spectra were analyzed by a Nicolet Nexus 870 spectrometer (Nicolet Instruments Inc. Madison, WI, USA). The zeta potential and dynamic light scattering (DLS) measurements were performed on a Malvern ZS90 Zetasizer Nano ZS instrument (Malvern, UK). N_2_ adsorption/desorption isotherms were determined by a Micromeritics Tristar 3000 analyzer (Micromeritics Instruments Corporation, Atlanta, GA, USA). The UV-vis-NIR absorption spectra were obtained on a UV-1800 spectrophotometer (Shimadzu, Japan). The drug loading (DL) ratio of PFD was calculated through the formula below (Equation 1):


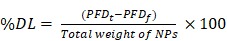
 (Equation 1)

where, PFD_t_ is the total quantity of PFD used in the synthesis of NPs and PFD_f_ is the quantity of unencapsulated PFD present in the supernatant.

### *In vitro* redox-triggered biodegradation of HMON-CHO

The *in vitro* biodegradation behavior of the HMON-CHO nanoparticles in PBS was investigated according to a previously reported protocol 25. A total of 2 mg of HMON-CHO nanoparticles were incubated in 5 mL of PBS at 37 °C under slow stirring. In order to mimic the intracellular and extracellular environments, 200 μL of GSH (10 mM) aqueous solution were added. At the given time intervals, HMON-CHO were taken, collected by centrifugation, and washed with PBS before performing TEM imaging and DLS detection. Also, the degradation percentages of HMON over time were determined by weighing the samples before and after two weeks of degradation.

### *In vitro* pH/GSH-triggered drug release

The *in vitro* PFD release of PFD@HMONs-Gem was studied *via* dialysis in different release media. Briefly, 5 mg of PFD@HMON-Gem particles were dispersed in 1 mL of buffer solution under different conditions. They were then injected into a dialysis bag (cutoff MW = 3500 Da) and dialyzed against 10 mL of PBS solution at pH 6.5 or pH 7.4 at different GSH concentrations of 0 and 10 mM, which was then shaked at 100 rpm at 37 ºC. At scheduled time intervals, 1 mL of each sample solution was removed and an equal amount of fresh PBS solution was added. The total amount of PFD released at each time interval was measured by UV-vis spectrophotometry at a wavelength of 263 nm. Meanwhile, the release of Gem was determined in a similar process (pH 6.5 or pH 7.4) and the concentration of released drug was analyzed by high-performance liquid chromatography (HPLC, Agilent 1200, USA). The chromatographic conditions for GEM were as follows: λ_max_ = 268 nm, mobile phase, methanol: aqueous ammonium acetate = 10: 90 (v/v) (pH adjusted to 5.7 with 0.1% (v/v) acetic acid).

### Cell culture

SW1990 cells were provided by the American Type Culture Collection (ATCC, Manassas, USA). Human umbilical vein endothelial cells (HUVEC) were obtained from the Chinese Academy of Sciences (Shanghai, China). SW1990 and HUVEC cells were cultured in a DMEM medium containing 10% FBS, penicillin (100 U/mL), and streptomycin (100 mg/mL). Human pancreatic stellate cells (PSCs) were purchased from ScienCell research laboratory (Carlsbad, CA), and cultivated in a specific stellate cell medium provided by the same supplier. All cells were maintained in a humidified atmosphere (95% R.H.) of 5% CO_2_ at 37 °C and cultured in the laboratory for less than 3 months after resuscitation; the established PSCs were used between passages 3 and 8.

### Cellular uptake analysis

To investigate the intracellular localization of the nanoparticles, confocal laser microscope observation (CLSM, Leica Microsystems, Mannheim, Germany) was performed. Briefly, SW1990 cells were seeded on a confocal dish (Cellvis, Califonia, USA). After cells were approximately 70% confluent, the medium was replaced with 2 mL of fresh medium containing 5 μg/mL of pure FITC or HMON-FITC (FITC and incubated for 1 h and 4 h. In the HMON-FITC + UTMD (HMON-FITC + U) group, a Sonopuls190 therapeutic ultrasound (US) unit was employed. The ultrasound transducer was placed at the bottom of the dish with coupling medium on the surface of the transducer. The amount of microbubbles and the condition of UTMD (1.2 W/cm^2^; duty cycle: 20%; microbubbles: medium 20 % (v/v); PRF 1MHz, 30s) were applied according to our previous study 28. Cells were then washed with PBS in triplicate, and treated with 50 nM Lyso Traker red for another 1 h to stain the lysosomes. Then, the cells were washed twice with PBS, and the nucleus was stained with DAPI for 15 min. Cells were further washed three times with PBS and the fluorescence images of the cells were then taken by confocal laser microscope.

For flow cytometry, SW1990 cells were seeded in a 6-well plate at a density of 3×10^5^ cells per well and incubated overnight. Treatment with free FITC or HMON-FITC for 1 h and 4 h at 37 ºC, was then performed. For the HMON-FITC + UTMD group, the same protocol was carried out as described above. The cells were then trypsinized and centrifuged for 3 min to remove excess FITC from the extracellular medium before ultimately being analyzed by flow cytometry (Accuri® C6, BD, New York, USA).

To further study the uptake and degradation behaviors of HMON in cells, a bio-transmission electron microscope (Bio-TEM, Hitachi HT7700, Tokyo, Japan) was employed. SW1990 cells were co-incubated with HMON nanoparticles (100 μg/mL in DMEM) for 1 h, 4 h, 4 d, and 7 d. For the HMON + UTMD group, a similar protocol was performed as the one used in CLSM experiment. After washing with PBS for three times, SW1990 cells were fixed with 2% glutaraldehyde for 2 h at room temperature, followed by 2 h in 1% OsO_4_. Each cell sample was then dehydrated through a graded ethanol series, embedded in EPOM812 and polymerized in an oven at 37 ℃ for 12 h, 45 ℃ for 12 h, and 60 ℃ for 48 h. All samples were examined by bio-TEM imaging.

### *In vitro* cytotoxicity and antitumor activity

A CCK-8 assay was carried out to assess the *in vitro* cytotoxicity of HMON nanoparticles. In brief, SW1990 and HUVEC cells were each seeded into a 96-well plate at 2×10^4^ cells per well, respectively. After a 24 h culture, cells were incubated with Gem or HMON-Gem (final Gem concentrations ranging from 0.1 to 1000 μg/mL). For the group of HMON-Gem with UTMD (HMON-Gem + U), the UTMD technique was performed as described above. Meanwhile, cells treated with PBS were used as a control group. After 24 h, 10 µL of CCK-8 solution was added to each well, followed by a 4 h incubation for at 37 ℃. The OD value was measured at 450 nm by a Spectra Max 190 microplate reader (BIO-RAD; Hercules, CA).

To investigate the pro-apoptotic effect of HMON-Gem on tumoral cells, SW1990 cells were seeded in a 6-well plate at a density of 5.0 × 10^5^ cells per well. When those cells reached a confluency of 70-80%, they were then exposed to the HMON-Gem nanoparticles at Gem concentration of 5.0 μg/mL. Cells treated with PBS were used as a negative control. After a 24 h incubation, the cells were collected and re-suspended in 500 μL of ice-cold binding buffer, followed by 5 μL of Annexin V-FITC and 5 μL of PI solution. The final samples were incubated in the dark at room temperature for 15 min prior to a flow cytometry analysis. This experiment was repeated in triplicate for each sample.

Further investigation to study the antitumor activity of HMON-Gem involved accessing the cellular apoptotic by the calcein-AM/PI double-labeling assay. SW1990 cells were seeded in a 24-well plate at 1×10^5^ cells per well and incubated at 37 ºC overnight. After treatment with 50 μg/mL of the aforementioned nanoparticles for 6 h, cells were washed with PBS for three times and cultured for another 24 h. The cells were subsequently stained by a mixture of Calcein-AM and PI solution for 10 min. Apoptotic cell death (red) and living cells (green) were measured under an inverted fluorescent microscope (Leica DMi8, Leica, Germany).

### Immunocytochemistry (ICC) analysis *in vitro*

For an ICC assay, PSCs and SW1990 were co-cultured into a 24-well plate and cultured at 37 °C for 24 h to allow for adhesion. Cells were incubated with PBS, free PFD (0.3 mg/mL PFD), PFD@HMON-Gem (0.3 mg/mL PFD), and PFD@HMON-Gem with pH 6.5 (achieved using acetyl acid and DMEM to alter pH; 0.3 mg/mL PFD), for 48 h. After treatment, cells were fixed with 4% paraformaldehyde for 15 min, followed by washing with PBS in triplicate. A Strept Avidin-Biotin Complex (SABC) Method was utilized to analyze the expression levels of regulatory components in the ECM. Briefly, cells were blocked with 5.0% BSA for 30 min prior to antibody labeling against either collagen I (rabbit, Abcam, ab34710) or fibronectin (rabbit, Abcam, ab 32419) 2 h at 37 ℃, respectively. Subsequently, cells were incubated with Strept Avidin-Biotin Complex (SABC) for 20 min, a color reaction was developed with a DAB detection system. Cells were then counterstained with hematoxylin for 1 min. Three horizons in the high-power perspective (200×) of each slice were taken randomly.

### Nude mice xenograft model

BALB/c nude mice (male, 6 weeks age, 17-20 g) were purchased from Sippr-BK laboratory animal Co. Ltd (Shanghai, China) and kept in the Laboratory Animal Center of Shanghai General Hospital (Shanghai, China). All animal protocols were approved by the Shanghai Jiaotong University Animal Care and Use Committee. To establish the pancreatic co-implanted tumor model, PSCs (3×10^6^ cells/mouse) and SW1990 (3×10^6^ cells/mouse) were subcutaneously co-injected into the dorsum above the right leg. Tumor nodules were examined weekly to analyze size as measured by a caliper and calculated using the following formula (Equation 2).


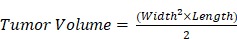
 (Equation 2)

### *In vivo* pharmacokinetics and fluorescence biodistribution

Evaluation of the blood circulation profile of PFD@HMON-Gem nanoparticles was performed as healthy mice (*n* = 3) were injected with PFD and PFD@HMON-Gem at the equal PFD dose (40 mg PFD/kg) through tail vein 9. At the designated time intervals post-injection (0.5, 1, 2, 3, 4, 8, 12, 16, 20 and 24 h), blood was collected from the ocular vein then dissolved in 300 µL of lysis buffer. The blood samples were centrifuged at 12000 rpm for 15 min and 4 ºC. The concentration of PFD was measured by UV-Vis spectrophotometry at 263 nm. Data are expressed as the percentage injected dose per gram blood (%ID/g) and fitted using the 3P97 software.

In order to investigate the biodistribution of PFD@HMON-Gem nanoparticles with UTMD treatment, DiR-labeled nanoparticles were injected intravenously into PSCs and SW1990 co-implanted tumors. Monitoring of the biodistribution was achieved using a Maestro *in vivo* fluorescence imaging system (CRi, Inc. Woburn, MA, USA). For the DiR-labeled HMON-Gem with UTMD (DiR@HMON-Gem + U) group, the mice received a simultaneous injection of 200 μL of microbubbles (5.9 mg SonoVue dissolved in 5 ml saline) from the retroorbital venous plexus. In these samples, UTMD was conducted at the tumor site according to our previous study 28. Real-time fluorescence imaging was performed at the time intervals of 0.5 h, 1 h, and 4 h. At 4 h after injection, major organs (heart, liver, spleen, lung, and kidney) and tumors were excised for *ex vivo* imaging.

Then, the *in vivo* metabolism process of PFD@HMON-Gem was estimated. PFD@HMON-Gem at the equal PFD dose (40 mg PFD/kg) was injected intravenously to SW1990 and PSCs co-implanted tumors. At varied time intervals (2, 6, 12, 24, 36, and 48 h), the urine and feces were collected and carried out by ICP-AES measurement to determine the Si content.

### Tumor immunohistochemistry (IHC) assays

For IHC analysis, nude mice bearing model tumors were treated with different formulation of PFD (free PFD, PFD@HMON-Gem, or PFD@HMON-Gem + UTMD) (PFD concentration: 40 mg/kg equiv.) *via* intravenous injection every 3 days (for a total of 7 injections). PBS was used as the control. After 3 weeks, all the tumors were harvested and fixed in formalin followed by paraffin embedding. The paraffin blocks were sectioned into 4 μm-thick slices, de-paraffinized, rehydrated, and boiled in 0.01 M citrate buffer (1000 mL) at pH 6.0 for 1 h. Staining of ECM associated proteins (*e.g.,* collagen I and fibronectin) was carried out using the Strept Avidin-Biotin Complex (SABC) Method, as previously described. Afterwards, tissue sections were counterstained with hematoxylin (Beyotime, China) for 1 min, cleared in xylene, and coverslipped. The samples were analyzed on a light microscope (Leica DMi8, Leica, Germany). Five independent horizons of each slice were examined randomly.

### Analysis of rhodamine penetration in tumor

Penetration of the nanoparticles was investigated using different formulations with or without PFD (Saline, PFD, PFD@HMON-Gem and PFD@HMON-Gem + UTMD) (PFD concentration: 40 mg/kg equiv.) were injected intravenously to SW1990 and PSCs co-implanted tumors. For the PFD@HMON-Gem + UTMD treated group, UTMD was performed as above. After three weeks, rhodamine was intravenously injected and subsequently, all the tumors were harvested and frozen *via* liquid nitrogen. The as-obtained slices (4 μm) were air-dried and fixed *via* formalin for 30 min. The nuclei were counterstained with DAPI. Sections were then washed with PBS for three times and coverslipped for imaging by confocal microscopy.

### Therapeutic effect *in vivo*

To examine the therapeutic effect of various formulations on a nude mice xenograft model of pancreatic tumor, different treatments were began when the tumor volume reached approximately 100 mm^3^. Mice were divided into five groups, at random (*n* = 6), and each group was administrated with one of the formulations: control (saline, 200 μL), free Gem (20 mg/kg, in 200 μL PBS), free PFD (40 mg/kg, in 200 μL PBS), PFD@HMON-Gem ([Gem] = 20 mg/kg, [PFD] = 40 mg/kg, in 200 μL PBS), and PFD@HMON-Gem + UTMD (PFD@HMON-Gem + U) ([Gem] = 20 mg/kg, [PFD] = 40 mg/kg, in 200 μL PBS) by intravenous injection in the tail vein 9. All of the xenograft nude mice received the injection every 3 days, for a total of 7 injections. For the PFD@HMON-Gem + U group, UTMD was performed simultaneously with the injection (2 min, 2W/cm^2^, 1MHz, 20% duty cycle, according to our previous study 28). The weight of each mouse was recorded every three days and the growth profile of the tumor size was observed twice a week. The survival time of each mouse was also recorded and evaluated by Kaplane-Meier Analysis.

### Blood biochemical indexes and histological examination

After 21 days of treatment, the representative mice from the five groups were sacrificed by carbon dioxide asphyxiation. Tumors and major organs including heart, liver, spleen, lung, and kidney were harvested and fixed in 4% formaldehyde for 48 h. Subsequently, the tissues were embedded in paraffin and sectioned into 4 μm slices, followed by staining with hematoxylin and eosin (H&E), and analysis under a light microscope to investigate pathological changes. Cell apoptotic profiles in the tumor sections were evaluated by staining with a terminal deoxynucleotidyl transferase dUTP nick end labeling (TUNEL) apoptosis assay kit, according to manufacturer's instructions.

Blood samples were collected from the eye vein after quickly removing the eyeball. After standing at 4 ºC for 3 h, the collected blood samples were centrifuged at 1000 rpm for 15 min to obtain serum. The blood biochemistry analysis was determined using a fully automatic biochemistry analyzer (ADVIA2400, Siemens, USA). The remaining animals of each group were kept to monitor the daily body weight change and the survival rate (*n* = 6).

### Statistical analysis

Every experiment was repeated in triplicate and the results are shown as mean ± standard deviation (S.D.). Statistical analysis was conducted by the Student's *t* test for 2 groups or one-way ANOVA for multiple groups, followed by Newman-Keuls test. A value of 0.05 was set as the significance level; the data were marked as (*) *P* < 0.05, (**) *P* < 0.01, and (***)* P* < 0.001.

## Results and Discussion

### Synthesis and characterization of PFD@HMON-Gem

**Figure 2:**
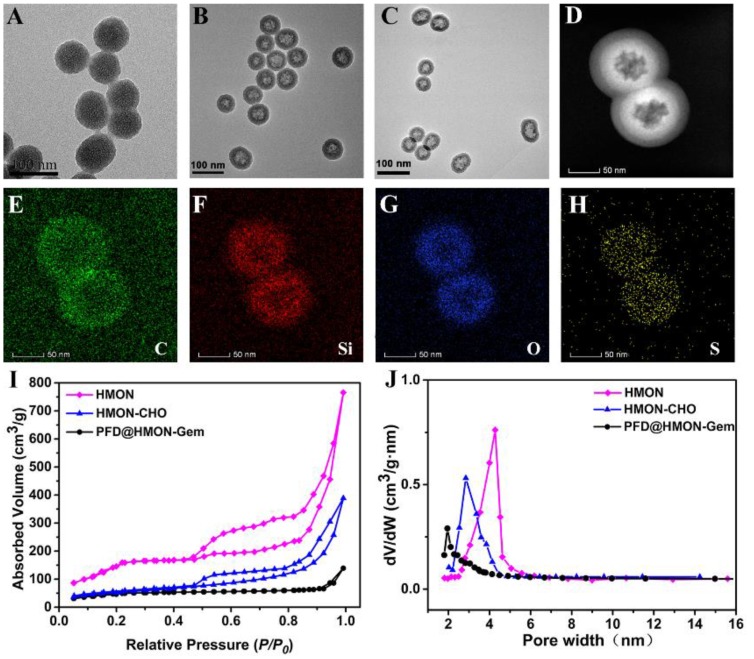
TEM images of (A) SiO_2_@MON, (B) HMON and (C) PFD@HMON-Gem. (D) dark-field TEM of HMON. (D-H) Element mapping of HMONs: (E) C, (F) Si, (G) O, and (H) S. (I) N_2_ adsorption-desorption isotherm and (J) the corresponding pore-size distribution of HMON, HMON-CHO and PFD@HMON-Gem.

The monodispersed HMON nanoparticles were synthesized according to a previous reported procedure 25, 36. In a “chemical homology” method, the core-shell structured SiO_2_@MON nanoparticles were prepared through the formation of organic-inorganic hybrid MONs onto the surface of SiO_2_ nanoparticles by the co-hydrolysis and co-condensation of TEOS and BTDS. It is worth noted that CTAC and TEA are used as the pore-generating agent and catalyst, respectively. By TEM observation, nearly monodispersed spherical SiO_2_@MON nanoparticles were fabricated with the diameter approximately 65 nm (Figure 2A). After etching away the SiO_2_ core in ammonia solution, the hollow structured MON frame (HMON) was formed with a slightly increased diameter of approximately 72 nm (Figure 2B). The elemental mapping images (Figure 2C-H) of HMON confirmed the homogeneous distribution of C, O, Si, and S elements. The N_2_ absorption-desorption isotherm of HMON exhibited the presence of a well-defined mesoporous structure with a large surface area of 814.3 m^2^/g and a uniform pore size of 4.27 nm (Figure 2I and J). The unique dimensions of the as-synthesized HMON nanoparticles satisfied those requirements of an effective delivery system for various payloads. Amino groups were then bonded to HMON nanoparticles in order to increase surface reactivity. The Fourier transform infrared (FT-IR) spectra (Figure S1) and zeta potential change (Figure S2) verified the successful amination of HMON (HMON-NH_2_). The amino functional groups on HMON nanoparticles then reacted with 4-formylbenzoic acid to form aldehyde-functionalized HMON-CHO nanoparticles. The successful completion of the benzaldehyde functionalization was confirmed by the C=O stretching vibration observed at 1661 cm^-1^ in FTIR and the zeta potential change (58.0 mV). With the series of modifications, the specific surface area of HMON-CHO decreased to 457.8 m^2^/g with the average pore size decreasing from 4.27 to 2.65 nm (Figure 2J).

**Figure 3:**
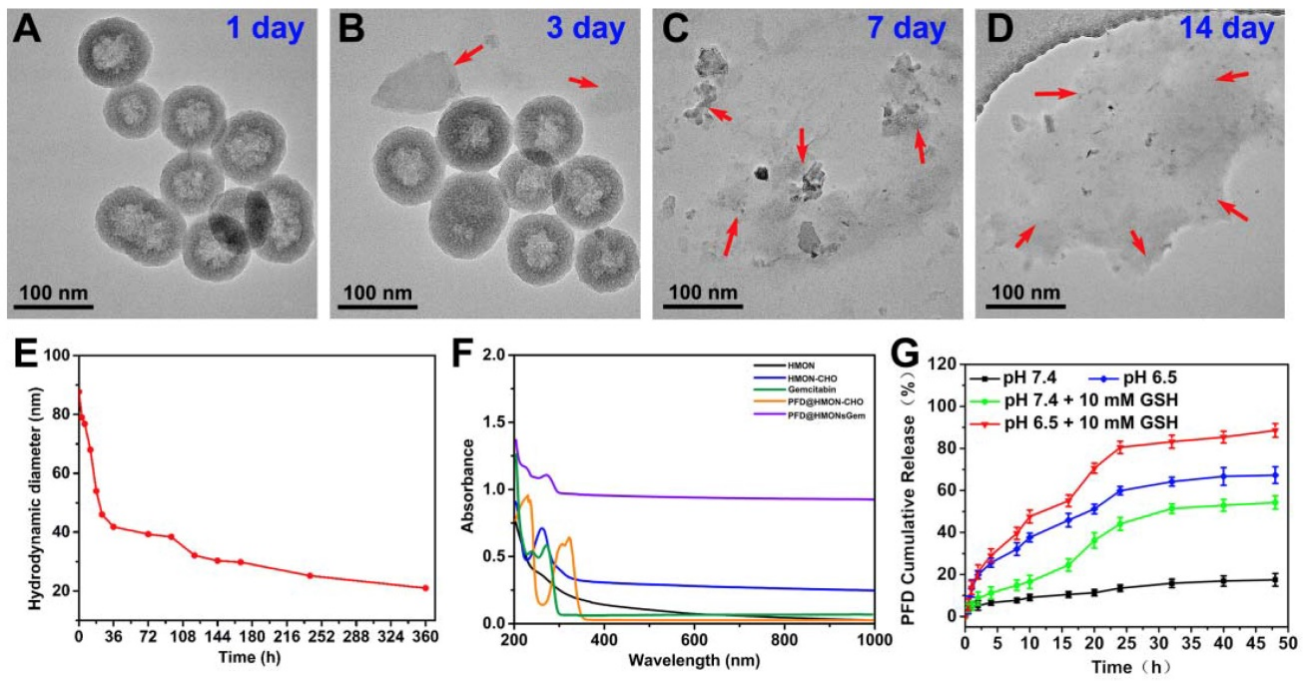
TEM images of HMON-CHO dispersed in PBS aqueous solution containing 10 mM GSH for (A) 1 day, (B) 3 day, (C) 7 day, and (h) 2 weeks. (E) DLS data curves of the HMON-CHO degradation process in PBS at GSH concentrations of 10 mM. (F) UV-vis-NIR spectra of HMON, HMON-CHO, free gemcitabine, PFD@HMON-CHO and PFD@HMON-Gem. (G) PFD release from PFD@HMON-Gem in PBS at pH 6.5 and 7.4 with or without adding 10 mM of GSH.

It has been proved that the disulfide bond can be cleaved in the reductive tumor environment 37. Thus, a similar redox responsive behavior was expected in the disulfide bond equipped HMON system of this study. As shown in Figure 3A-D, HMON was found to degrade gradually as a function of incubation time in a GSH (10 mM) containing PBS solution. After 14 days, degradation was considered complete upon observing no sign of the presence of the regular spherical HMON particles. Correspondingly, the degradation percentages of HMON-CHO were determined in GSH (10 mM) containing PBS solution by weighing the nanomaterials before and after different time of degradation, which were calculated to be of 77.3% and 89.2% after 7 and 14 day biodegradation, respectively (Figure S3). In addition, the biodegradation behavior of HMON-CHO was explored in GSH (10 mM) containing SBF solution at elevated concentrations (0, 5, and 10 mM). As shown in TEM images (Figure S4), HMONs-CHO in SBF without GSH exhibits feeble biodegradation of nanostructure during 7 d observation. In particular, the biodegradation rate was significantly accelerated when it was incubated with PBS at elevated GSH concentrations (Figure 3A-D). The hydrodynamic diameter of HMON nanoparticles decreased accordingly from 90 nm to 20 nm in the mild reductive medium (GSH: 10 mM) (Figure 3E). Given the high GSH concentration (1-11 mM) in cancer cells, the favorable biodegradability of HMON nanoparticles observed here is regarded as a positive indication for bio-safety.

Next, the HMON-CHO nanoparticles were loaded with the antifibrosis drug, PFD. The change in zeta potential, as seen in Figure S2, along with the appearance of a new peak at 265 nm in the UV-Vis spectrum of PFD@HMON in Figure 3F presented strong evidence of a successful loading. Loading capacity of HMON-CHO (wt.% relative to the initial mass) was further calculated to be 35.1% as determined by UV-Vis spectrophotometry at 265 nm. In the final step, the antitumor drug Gem was attached to the HMON nanoparticles as a gatekeeper *via* a chemical reaction between the hydroxyl group of Gem and the benzaldehyde group on the PFD@HMON particles. Compared to initially synthesized HMON, the PFD loading and Gem capping modifcation showed negligible change of morphology and uniform mesoporous structure, which still maintained desirable monodispersity as demonstrated by TEM images (Figure 2C). The positive zeta potential confirmed a successful capping of Gem onto PFD@HMON (Figure S2). As expected, both pore size and surface area were evidently decreased after loading of PFD and Gem (Figure 2I-J). The hydrodynamic size of the final PFD@HMON-Gem particle increased to 115 nm as compared to 90 nm for the HMON particle alone (Figure S5). Meanwhile, no distinct changes of hydrodynamic size of nanoparticles were observed over 7 days, demonstrating the good colloidal stability of our fabricated PFD@HMON-Gem nanoparticles (Figure S6).

### *In vitro* pH/GSH-triggered PFD release

Intractable problems remain challenges for many gatekeeper materials with regard to their biosafety and bio-responsiveness 38, both critical factors determining their *in vivo* potential. Development of biocompatible, non-toxic, gatekeeper materials with favorable stimuli-responsive properties to adapt to the complex tumor microenvironment creates excitement in the field. In the work presented herein, a biocompatible Gem molecule was used as a pH-sensitive “gatekeeper” to avoid any exogenous capping agents.

The release rate of PFD from the PFD@HMON-Gem was investigated under different pH conditions *in vitro*. The gating effect of the Gem molecule was first evaluated as compared to the control of the PFD@HMON without the Gem gatekeeper. It was clearly observed that Gem capping played an effective role on controlling the release of PFD from the HMON system. At pH 7.4 over the course of 8 h, the PFD release rate was less than 20% for PFD@HMON when capped with Gem (Figure 3G). Experimentation showed that Gem capping release profiles were significantly lower than those for PFD@HMON without capping (> 70 wt.% at pH 7.4 and > 84 wt.% at pH 6.5) over the same time period (Figure S7). It was also found that the drug release profile of PFD@HMON-Gem was highly pH dependent. The cumulative release rate of PFD increased to 67.3% after 48 h at pH 6.5. The acidic environment was believed to trigger the cleavage of the acetal covalent bond between gatekeeper Gem and HMON particles, thus increasing the PFD release. Also, the released PFD was sharply increased to ≈ 54% and ≈ 88% at pH 7.4 and 6.5 over a span of 48 h in the presence of GSH. The GSH-triggered PFD release from PFD@HMON-Gem nanoparticles is mainly attributed to the hydrolyzation of disulfide bond within the framework under the reducing condition. Interestingly, a pH-triggered Gem release behavior was observed (Figure S8), mainly due to cleavage of the acetal bond in acid medium, resulting in the breakage of connection between Gem and HMON. Given the fact that a tumor extracellular matrix microenvironment is mildly acidic 25, this drug-gated strategy should regulate a controlled release of different payloads to targeted tumor extracellular matrices.

### ECM down-regulation *in vitro*

**Figure 4:**
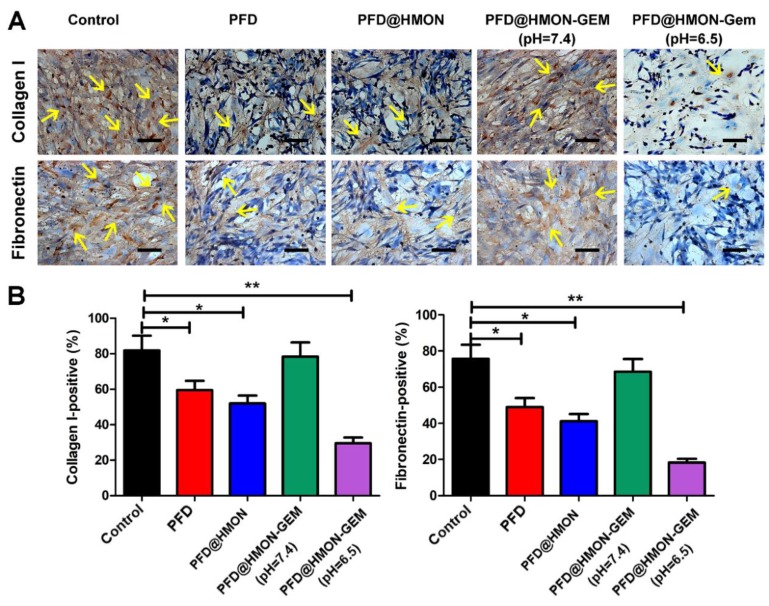
The effects of PFD@HMON-Gem on the key components of ECM *in vitro*. (A) Expression levels of collagen I and fibronectin after incubation with various PFD formulations (PFD concentration: 0.3 mg/mL) detected by ICC measurement. Scale bars: 50 μm. (B) Quantificative analysis of collagen I/fibronectin-positive staining areas in each group (mean ± SD, *n* = 5), * *P* < 0.05, ** *P* < 0.01 as compared to control group.

As the pivotal stromal components secreted by PSCs, collagen I and fibronectin constitute the essential architecture of the ECM providing tensile strength, mechanical buffering, and contributing to neoplasia and metastasis 3, 6, 26, 39-42. Hence, collagen I and fibronectin were selected as model proteins by which to evaluate the suppression efficacy of each PFD formulation on ECM. As seen in Figure 4, after treated with different formulations of PFD (0.3 mg/mL PFD equiv.) for 48 h, the number of cells staining positive for both collagen I and fibronectin decreased significantly in PFD@HMON and free PFD treated groups as compared to the control. The down-regulation effect of PFD@HMON-Gem on collagen I and fibronectin were likely compromised due to Gem molecules effectively sealing the pores on the HMON nanoparticles. Disruptions in pore openings thereby inhibited the PFD release rate (Figure 3G) at pH = 7.4. In comparison, the ECM expression (represented by collagen I and fibronectin levels) was successfully down-regulated to the level similar to those of the free PFD and PFD@HMON groups at pH = 6.5. These results demonstrated the release rate of PFD can be effectively controlled in response to low pH (*e.g*., tumoral micro-environment) with nanoparticles capped with pH-responsive Gem as a gatekeeper, which may eventually enable down regulation of ECM expression around carcinomas.

### Cell uptake assay

**Figure 5:**
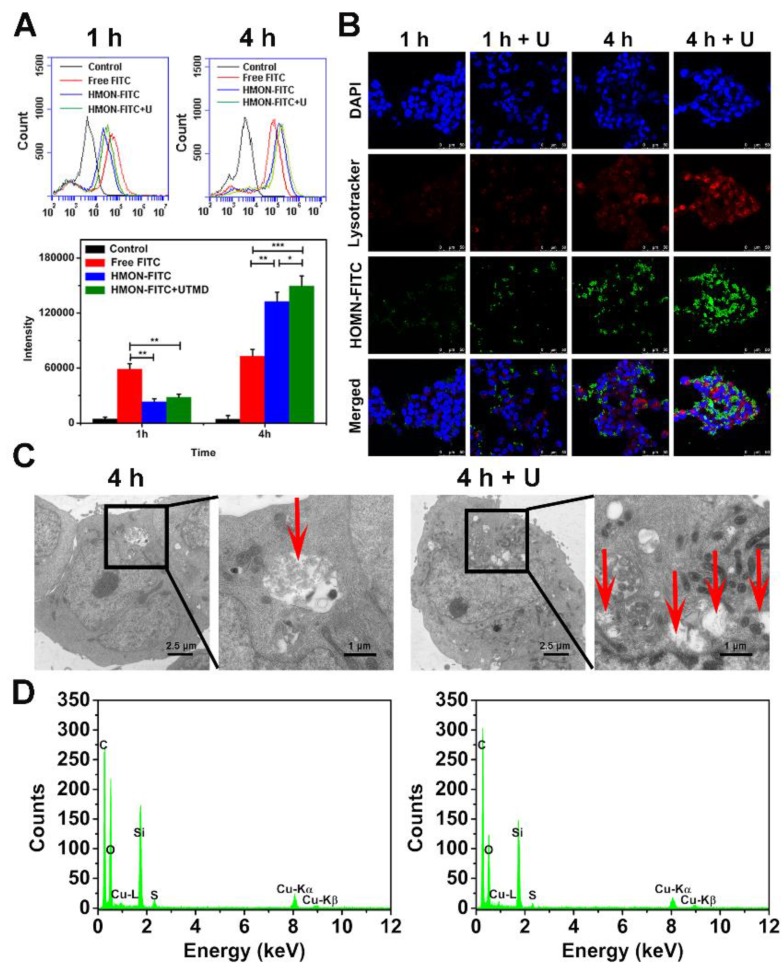
*In vitro* cellular uptake of free FITC, HMON-FITC and HMON-FITC + UTMD at different time points. (A) Flow cytometry for cells treated with free FITC, HMON-FITC with or without UTMD for 1 h or 4 h. (B) Confocal laser scanning microscope images of SW1990 cells co-cultivated with free FITC, HMON-FITC with or without UTMD at different time periods (green, FITC; red, Lysotracker; blue, DAPI, scale bar = 50 μm). (C) Representative bio-TEM images of SW1990 cells and (D) the corresponding energy dispersive spectrometer (EDS) analysis after incubated with HMON with or without UTMD for 4 h. Box areas and red arrows clearly show the microstructure of HMON intracellularly.

As shown in Figure 5A, the FITC fluorescence intensity of cells in the HMON-FITC group and HMON-FITC + U group was less than that of free FITC after 1 h of incubation. Decreased fluorescence intensity was likely due to the slow internalization of HMON-FITC by cells through the endocytosis process. After 4 h, the measured intensity was much higher for those cells treated with HMON-FITC or HMON-FITC + U than those with free FITC. This observation was in an agreement with a previous report claiming that nanocomposite delivery enhanced the cell update of FITC 28. Under the confocal laser scanning microscope (Figure 5B), relatively weak FITC fluorescence in the HMON-FITC group was observed at 1 h. However, after 4 h, cell uptake was significantly improved, particularly with the assistance of UTMD. A mild UTMD resulted in an increase in the number of pores and clathrin, thus improving the intracellular delivery of this nanoplatform. It is worth noted that there was no significant difference in cell uptake between HMON and HMON + U samples at 1 h. This result was attributed to the effective ease of nanoparticle penetration of the larger cells in the cell matrix from the flow cytometry experiment as compared to the smaller, stabilized cells in the confocal assay.

The enhanced cell uptake of the HMON system with UTMD was also confirmed by bio-TEM images. As shown in Figure 5C, only a limited number of HMON particles were observed within the cytoplasm after 4 h. With the UTMD treatment, cells treated with the same HMON showed a greater number of endocytic vesicles. This observation was consistent with our previous study in that UTMD increased internalization through endocytosis and accumulation inside cells 35. Interestingly, a 4 day incubation in cells resulted in structural collapse and dissolution of HMON nanoparticles (Figure S9), further confirming effective biodegradability of HMON nanoparticles previously shown in Figure 3.

### *In vitro* biocompatibility and therapeutic efficacy

**Figure 6:**
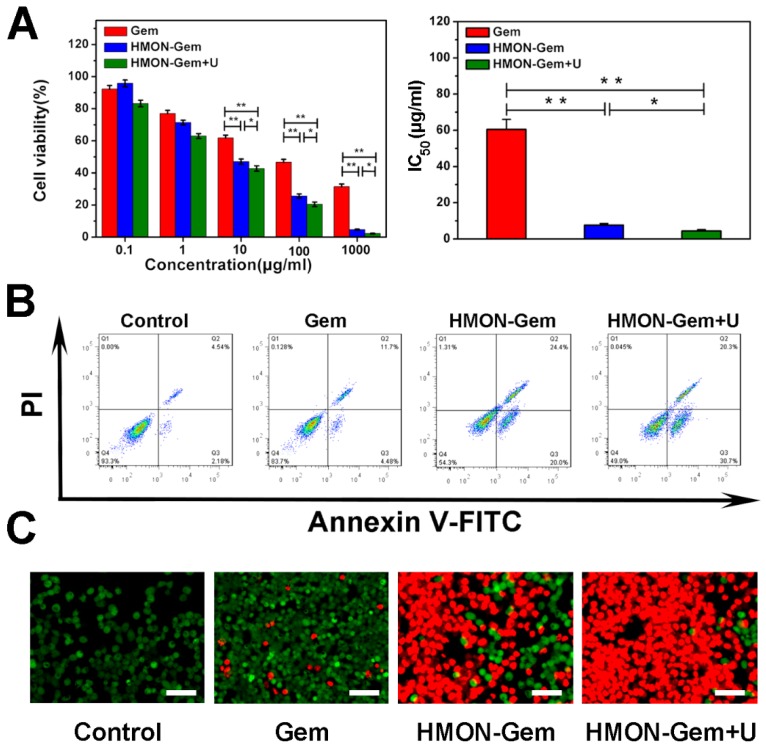
* In vitro* antitumor assays: (A) Cell viabilities of SW1990 cells incubated with Gem, HMON-Gem and HMON-Gem + U nanoparticles with UTMD at various concentrations (with equivalent concentrations of Gem, *n* = 6, * *P* < 0.05, ** *P* < 0.01 as compared to free Gem group). In the HMON-Gem + U group, the cells were also treated with UTMD and then incubated for 24 h. (B) Flow cytometric analysis of the apoptosis of SW1990 cells treated with different nanoparticles with or without UTMD and incubated for 48 h. (C) Fluorescence images of calcein-AM (green)/PI (red) double stained (bar = 100 μm).

Excellent biocompatibility of HMON was confirmed with no observed cytotoxicity to human healthy HUVEC cells (Figure S10). Cell viability remained nearly 100% at HMON concentrations up to 100 μg/mL in the CCK-8 assay. Viability remained above 80% at a very high concentration of 1000 μg/mL of HMON. Meanwhile, the CCK-8 assay was further employed to investigate the *in vitro* therapeutic efficacy of various Gem formulations against SW1990 pancreatic cancer cells. As seen in Figure 6A (left), the viability of SW1990 cells decreased as a function of Gem concentration in all three groups. Given the relatively low cytotoxicity of HMON, the lethality of HMON-Gem against SW1990 cells was considered significant. At Gem concentrations of 10 µg/mL, more than 50% of cells were killed in the HMON-Gem group and the HMON-Gem + UTMD groups. The fatality ratio was even higher when 100 μg/mL free Gem was applied. The targeted antitumor effectiveness of the HMON-Gem system was further confirmed by their half maximal inhibitory concentrations (IC_50_) at 24 h. As expected, the IC_50_ of either the HMON-Gem group (7.66 ± 0.86 μg/mL) or the HMON-Gem + UTMD group (4.43 ± 0.74 μg/mL) was significantly lower than that of the free Gem group (60.49 ± 5.50 μg/mL) (*P* < 0.01). The comprehensive data set demonstrated that the HMON delivery system had the potential to enhance the chemotherapeutic effect of Gem against pancreatic cancer cells. Moreover, the HMON-Gem + UTMD group resulted in decreased cell viability compared to the HMON-Gem group at all concentration levels, indicating the effective role UTMD played on improving the anti-proliferative effect 30, 43.

An in depth investigation on the pro-apoptotic mechanism of the HMON nanoparticles included flow cytometry analysis and calcein-AM/PI double staining. Figure 6B displays the apoptotic profiles of SW1990 cancer cells after treatment with different Gem formulations. The apoptotic rates of HMON-Gem group (45.7 %) and HMON-Gem + UTMD group (51.0 %) were greatly improved when compared to the free Gem group (16.3 %). The results clearly demonstrated the enhanced apoptotic and cytotoxic effects of the HMON-Gem drug system against pancreatic cancer cells, particularly when paired with the UTMD technique. This observation was further confirmed by the fluorescence images of calcein-AM/PI double stained samples (Figure 6C). Stained images resulted in increased concentrations of apoptotic cells (red labelled) in the HMON-Gem treated group compared to the free Gem treated group.

### *In vivo* bio-distribution and pharmacokinetics

**Figure 7:**
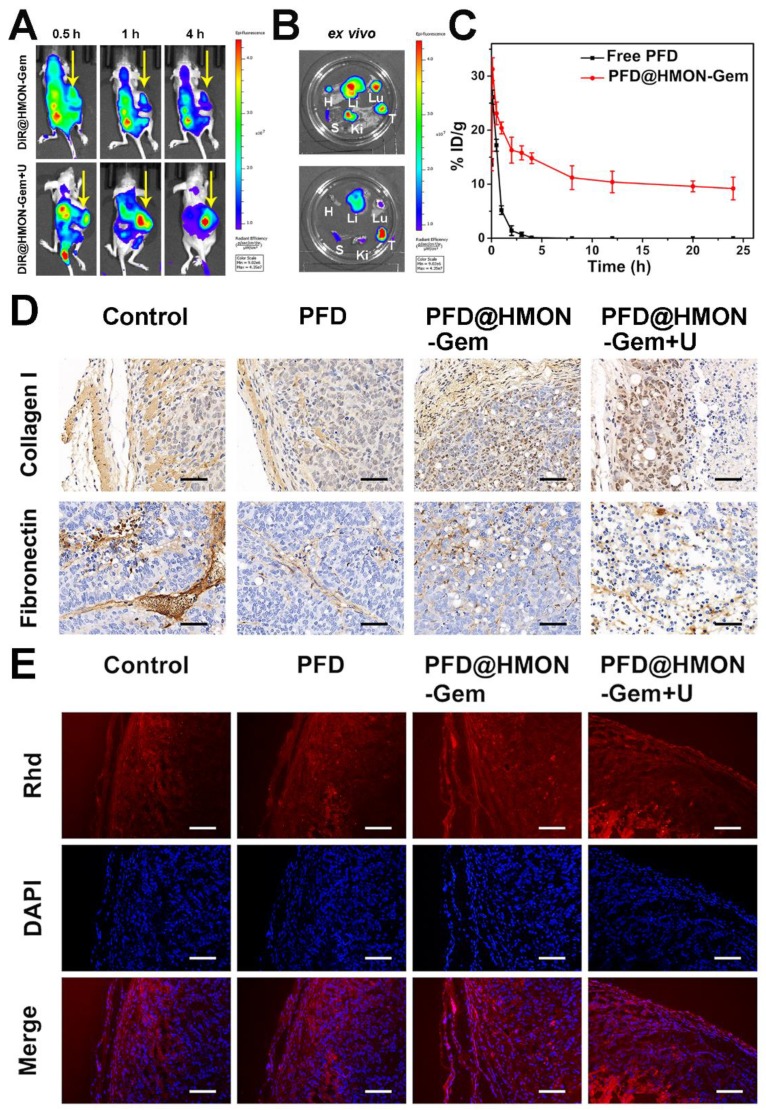
*In vivo* Bio-distribution and Pharmacokinetics**:** (A) Dynamic fluorescence biodistribution of DiR@HMON-Gem at different time intervals of 0.5 h, 1 h and 4 h after treatment with DiR@HMON-Gem and DiR@HMON-Gem + UTMD, respectively. Yellow arrows indicate the tumor foci of mice. (B) *Ex vivo* DiR fluorescence images of tumors and major organs (Li: liver, S: spleen, Ki: kidney, Lu: lung, H: heart and T: tumor). (C) *In vivo* pharmacokinetics of PFD@HMON-Gem in mice. Concentration of PFD in plasma is expressed as injected dose per gram of tissue (%ID/g). Data are presented as mean ± S.D. (*n* = 3). (D)The effects of PFD@HMON-Gem on the key components of ECM *in vivo*. (E) Evaluation of Rhd penetration: Rhd penetration and distribution in pancreatic tumor (SW1990/ PSCs co-implanted) tissues after 3 weeks' treatment of the different PFD formulations. Frozen tumor sections were stained with DAPI (blue) to label nuclei. Red: Rhd. Scale bars, 50 μm.

Inspired by the promising results of *in vitro* and *in vivo* biodistribution and accumulation of HMON nanoparticles in tumors, pancreatic tumor xenograft-bearing mice were investigated using the *in vivo* fluorescence imaging system. As shown in Figure 7A, the DiR fluorescence was only observed in the liver and/or kidney at 0.5 h post-injection for both cases (*i.e.,* with and without UTMD). The fluorescence signal in the tumor site increased slowly, reaching a maximum after 4 h in the DiR@HMON-Gem + UTMD group. For the DiR@HMON-Gem group, the fluorescence intensity of DiR accumulated in kidney was higher than that in tumor. Opposite distribution was observed in the DiR@HMON-Gem + UTMD group. This UTMD improved efficiency was in good agreement with the afore-mentioned *in vitro* cellular uptake results, as related to the effect of sonoporation 28, 44. After 4 h post-injection, the cancer cell bearing mice were sacrificed and major organs including heart, liver, spleen, lung, kidney and tumor were harvested for *ex vivo* imaging. Similar to the observation in the dynamic fluorescence images, the tumors of mice treated with DiR@HMON-Gem + UTMD exhibited a superior fluorescence intensity as compared to the group without UTMD (representative images for each group are shown in Figure 7B, and the other images are shown in Figure S11). These results along with the statistical analysis (Figure S12) further indicated a high tumor uptake of PFD@HMON-Gem nanoparticles, particularly with the assistance of the UTMD technique. In addition, we also collected the feces and urine of mice after intravenous injection to evaluate the *in vivo* metabolism of PFD@HMON-Gem nanopparticles, both feces and urine showed highest Si amount after 36 h post administration (Figure S13), thus suggesting the effective clearance of PFD@HMON-Gem by the way of feces and urine. The easy excretion of PFD@HMON-Gem out of the body is owing to the facile biodegradation of these nanoparticles.

The *in vivo* pharmacokinetics of PFD@HMON-Gem in mice was also evaluated (Figure 7C). Interestingly, the PFD@HMON-Gem system resulted in a much longer circulation time than free PFD. After a 3 h injection of free PFD, the concentration of PFD in the plasma quickly dropped to an undetectable level while 10% ID remained, even after 24 h post-injection of PFD@HMON-Gem. The slower release was attributed to the gatekeeping effect of attached Gem on the PFD@HMON nanoparticle system in this mild acidic cancer cell micro-environment. Our results demonstrate the benefits of the prolonged *in vivo* circulation time of the payloads.

### ECM down-regulation *in vivo*

Previously, the down-regulation effect of the PFD formulation on ECM was observed *in vitro*. Similarly here, tumor tissue sections were used to study the *in vivo* down-regulation of ECM. Specifically, different formulations of PFD were intravenously injected once every 2 days, (PFD dose: 40 mg/kg equiv.) into the SW1990/PSCs tumor bearing mice for a total of 3 weeks. Tumors were then excised and sectioned into 4 μm slices for IHC analysis. The results (Figure 7D and the statistical analysis was given in Figure S14) showed that, compared with control and free PFD treated groups, the expression levels of two major ECM components were down-regulated in PFD@HMON-Gem treated tumors. Further enhanced suppression efficiency of PFD@HMON-Gem with UTMD was observed. Alternatively, various degrees of cell necrosis were discovered in both the PFD@HMON-Gem and the PFD@HMON-Gem + UTMD group due to the presence of Gem. Although our results indicated suppressed expression of collagen I and fibronectin in the free PFD group, its efficacy was severely limited. This was attributed to its short circulation time and low tumor accumulation efficiency. Furthermore, the amount of nanoparticles accumulated in the PFD@HMON-Gem + UTMD group was similar to those results of the PFD@HMON-Gem group* in vivo*. The superior ECM suppression efficacy in the PFD@HMON-Gem + UTMD group was likely due to the enhanced permeability of the tumor vasculature by UTMD 43.

### Improvement of drug perfusion *in vivo*

It is well known that cancerous cells are commonly surrounded with a dense ECM lacking an avascular network, which resists the penetration of medicine into the tumor cells minimizing antitumor effectiveness 5, 10, 45. In this work, the anti-fibrosis drug, PFD, was pre-loaded into the HMON-Gem framework to inhibit the development of fibrosis (the main cause of ECM densification). Together with the UTMD technique used increase the intravascular osmotic pressure in such a way as to enhance the perfusion of drug, we aimed to ultimately improve the therapeutic efficiency of PFD@HMON-Gem. To this end, the fluorescence dye Rhd was used to visualize the tumor penetration of the HMON drug system *in vivo*. Tumor-bearing mice were supplemented with different formulations of PFD (dose: 40 mg/kg) *via* intravenous injection every 3 days. After 3 weeks, tumors were excised and analyzed for drug penetration profiles. As seen in Figure 7E, all tumor tissues showed a gradient fluorescence of Rhd from the edge of the tumor to the core. It is worth noting that the fluorescence intensity of Rhd was stronger in PFD@HMON-Gem group than that observed for the free PFD treated group or control. Further, the PFD@HMON-Gem + U group displayed even stronger fluorescence intensity of Rhd suggesting a deeper penetration (Figure S15). Previous studies indicated that UTMD could transiently increase the capillary permeability of normal and tumor tissues 29, 43, 44, 46. Under certain acoustic pressures, the resultant microbubbles created micro-jets to increase the endovascular osmotic pressure 47-49 and facilitate nanoparticle penetration 50. A similar mechanism could explain the improved PFD@HMON-Gem perfusion, as observed here.

### Therapeutic efficacy of tumor *in vivo*

**Figure 8:**
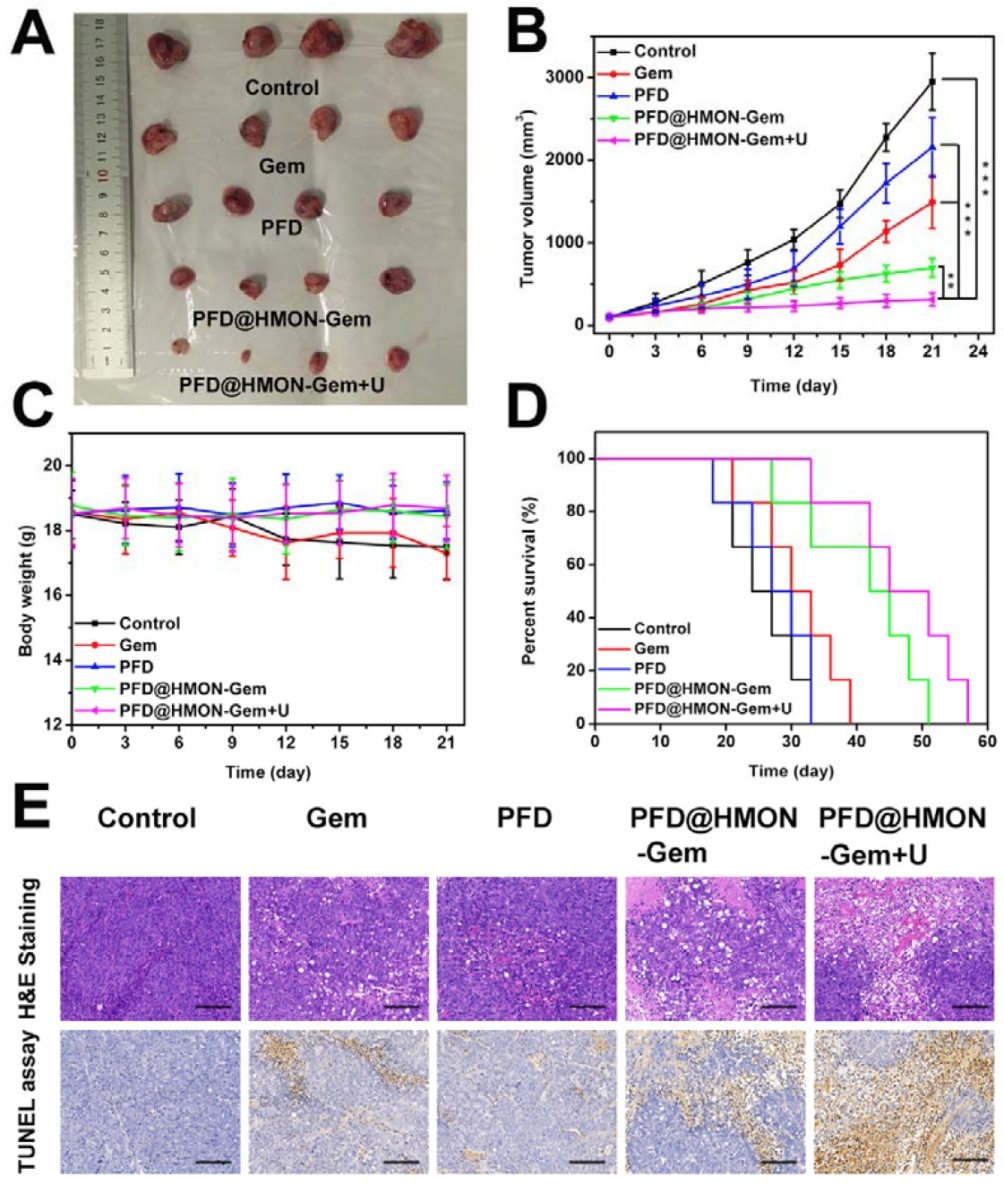
Therapeutic efficacy of control (saline), Gem, PFD, PFD@HMON-Gem, PFD@HMON-Gem + UTMD *in vivo*: (A) Subcutaneous tumor specimens at the end of the 21 day treatment; (B) tumor volumes over the treatment course in various groups (*n* = 6, **P < 0.01 and ***P < 0.001); (C) the average body weights of mice after various treatments; (D) Kaplan-Meier survival analysis of mice with tumors (*n* = 6); (E) H&E and TUNEL staining of tumor sections (bars = 100 μm).

The effective down-regulation of ECM and augmented endovascular osmotic pressure by UTMD may also increase the flux of PFD@HMON-Gem and therefore its therapeutic efficacy. To verify this hypothesis, the SW1990/PSCs pancreatic tumor-bearing mice were administrated with different formulations of Gem or free PFD. Firstly, the *in vivo* therapeutic efficacy was investigated by a direct measurement of tumor size. Pronounced inhibition of tumor growth was confirmed by photographs taken at day 21 of the tumors associated with those mice treated with PFD@HMON-Gem *+* UTMD. The tumor growth in other groups was moderately inhibited as compared to the control (Figure 8A). The average volume of the excised tumor measured at day 21 was 2949 ± 345 mm^3^, 697 ± 112 mm^3^, and 311 ± 79 mm^3^ for the control, the PFD@HMON-Gem and PFD@HMON-Gem + UTMD groups, respectively (Figure 8B). The inhibition effect was attributed to a combination of down-regulated ECM components and increased intravascular osmotic pressure induced by UTMD.

In addition, there was no substantial weight loss observed in any of the mice groups investigated, indicating good biosafety of the HMON nanoparticles (Figure 8C). Longer survival periods were observed for those mice treated with PFD@HMON-Gem, especially those receiving combined UMTD treatment (Figure 8D). The tumor apoptosis *in vivo* was also analyzed by H&E and TUNEL staining. The H&E and TUNEL stained tumor tissues of the PFD@HMON-Gem + UTMD group exhibited remarkable nucleus atypia and increased necrotic cells when compared to other groups (Figure 8E). Quantitative analyses of the H&E and TUNEL data are displayed in Figure S16, and also demonstrate the most significant effect of PFD@HMON-Gem + UTMD group act to both induce apoptosis and inhibit cell proliferation in tumor tissues. Overall, the PFD@HMON-Gem system particularly with UTMD improved the therapeutic efficacy for pancreatic tumors in nude mice, demonstrating a great potential in chemotherapy applications with positive biocompatibility and bio-safety.

### Systemic toxicity

After treatment with various formulations for 3 weeks, major organs of the mice were excised and sectioned for histological analysis (H&E staining) to study systemic toxicity. As shown in Figure S17, no sign of dysfunctional pathologies such as inflammation, injury, and necrosis were observed. These results confirmed the low, long-term toxicity of the HMON nanosystems. The systemic toxicity was further examined *via* blood analysis in which several routine serum biochemical indicators were measured (Figure S18). Considering the major side effects associated with blood indicators, no significant difference was observed between the control group and the PFD@HMON-Gem groups. The results of blood biochemistry also demonstrated that no liver or kidney toxicity was induced by PFD@HMON-Gem nanoparticles, suggesting their biosafety when used in cancer therapy.

## Conclusion

In this work, we designed a novel HMON system with excellent biodegradability and biocompatibility utilized for encapsulating and delivering PFD drug for pancreatic cancer treatment. The controlled release of the PFD drug was achieved through attaching a gating chemotherapeutic Gem to the HMON framework *via* chemical reaction. In a mild acidic environment, typically induced by cancer cells, the gating molecule Gem detached from the HMON nanoparticles *via* the cleavage of an acetal covalent bond. This cleavage allowed for the slow release of the PFD that eventually down-regulated the complex components of cancerous ECM. In addition, UTMD was employed as a powerful tool to enhance the intravascular osmotic pressure, thus increasing the penetration and accumulation of HMON nanoparticles in the tumor tissues. With the approved therapeutic efficacy and low systemic toxicity of HMON in the SW1990/PSCs pancreatic tumor-bearing mice, the present system shows great potential as a carrier to deliver nanomedicines with controlled release for effective cancer treatment.

## Supplementary Material

Supplementary figures and table.

## Abbreviations

ECM: Extracellular matrix
HMON: Hollow mesoporous organosilica nanoparticle
PFD: Pirfenidone
Gem: Gemcitabine
UTMD: Ultrasound target microbubble destruction
PSCs: Pancreatic stellate cells
MSNs: Mesoporous silica nanoparticles
FITC: Fluorescein isothiocyanate
IC_50_: Half maximal inhibitory concentration
TUNEL: Terminal deoxynucleotidyl transferase-mediated deoxyuridine triphosphate nick-end labeling
GSH: glutathione
CCK-8: Cell counting kit 8
