# Supplementary material for: Biodegradable, pH-Sensitive Hollow Mesoporous Organosilica Nanoparticle (HMON) with Controlled Release of Pirfenidone and Ultrasound-Target-Microbubble-Destruction (UTMD) for Pancreatic Cancer Treatment
Source: Theranostics. 2019 Aug 14;9(20):6002–18. doi: 10.7150/thno.36135 (PMC6735371; doi:10.7150/thno.36135)
Supplement: Supplementary file 1 — Supplementary figures and table. [file thnov09p6002s1.pdf]

Biodegradable, pH-Sensitive Hollow Mesoporous Organosilica Nanoparticle (HMON) with Controlled Release of Pirfenidone and Ultrasound-Target-Microbubble-Destruction (UTMD) for Pancreatic Cancer Treatment

Feng Gao[1#], Jianrong Wu[2#], Shiwei Niu[2#], Ting Sun[1], Fan Li[1], Yun Bai[1], Lifang Jin[1], Lizhou Lin[1], Qiusheng Shi[1✉], Li-Min Zhu[2✉], Lianfang Du[1✉]

1. Department of Ultrasound, Shanghai General Hospital, Shanghai Jiaotong University School of Medicine, Shanghai 201600, P.R. China.

2. College of Chemistry, Chemical Engineering and Biotechnology, Donghua University, Shanghai 201620, P.R. China.

Address for correspondence:

Lianfang Du, Department of Ultrasound, Shanghai General Hospital, Shanghai Jiaotong University School of Medicine, Shanghai 201600, P.R. China.

Email: du_lf@shsmu.edu.cn

Li-Min Zhu, College of Chemistry, Chemical Engineering and Biotechnology, Donghua University, Shanghai 201620, P.R. China.

Email: lzhu@dhu.edu.cn

Qiusheng Shi, Department of Ultrasound, Shanghai General Hospital, Shanghai Jiaotong University School of Medicine, Shanghai 201600, P.R. China.

Email: sqs19631989@163.com

Author Contributions

#: These authors contributed equally to this work.

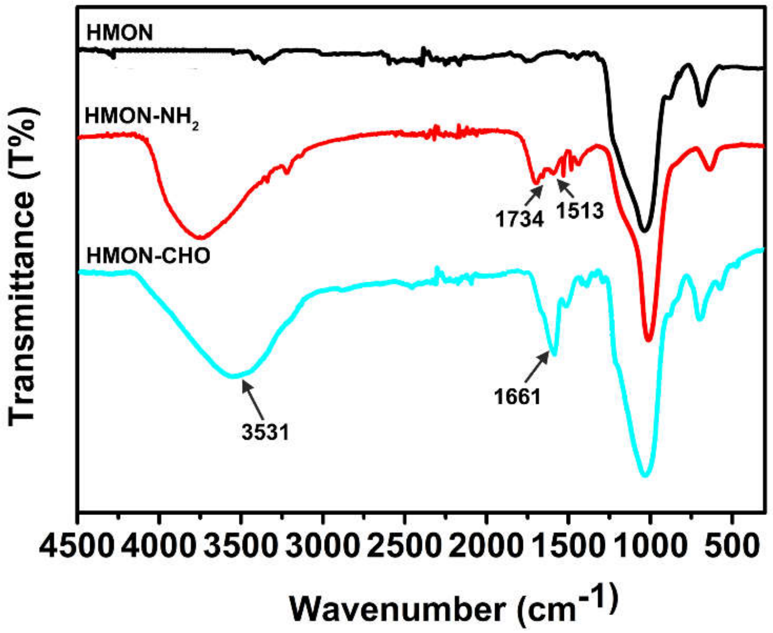

Figure S1. FT-IR spectra of HMON, $HMON-NH_2$ and HMON-CHO.

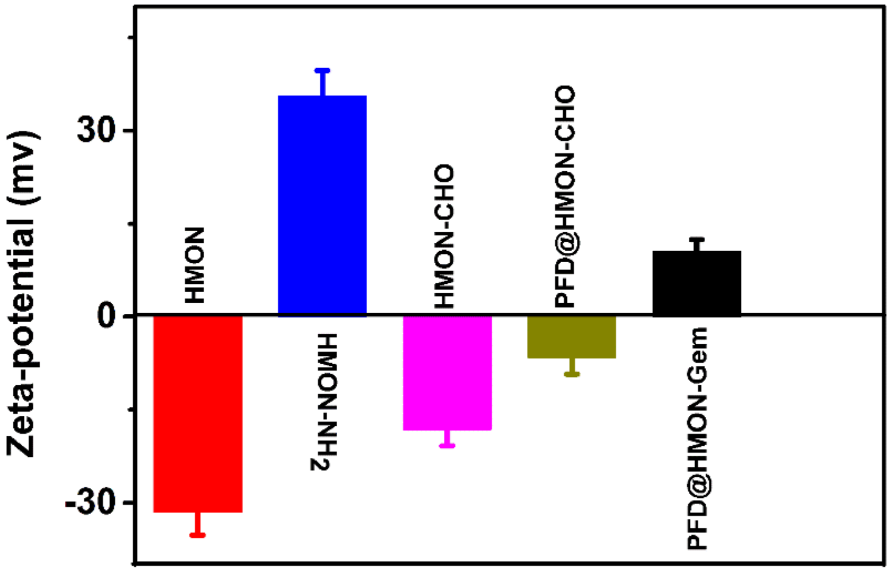

Figure S2. Zeta potentials of HMON, $HMON-NH_2$, HMON-CHO, PFD@HMON-CHO and PFD@HMON-Gem nanoparticles.

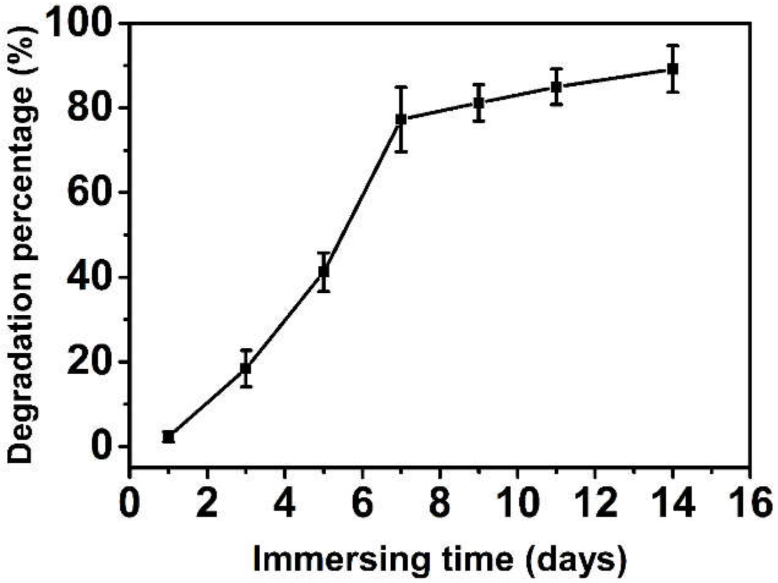

Figure S3. The degradation percentages of HMON-CHO over time were determined in GSH (10 mM) containing PBS solution.

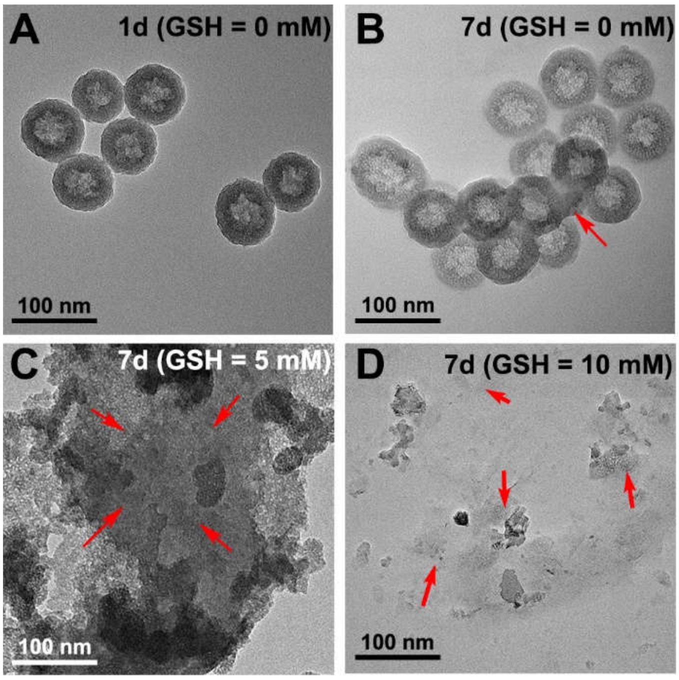

Figure S4. TEM images of HMON-CHO nanoparticles dispersed in in GSH containing PBS solution at elevated concentrations (0, 5, and 10 mM).

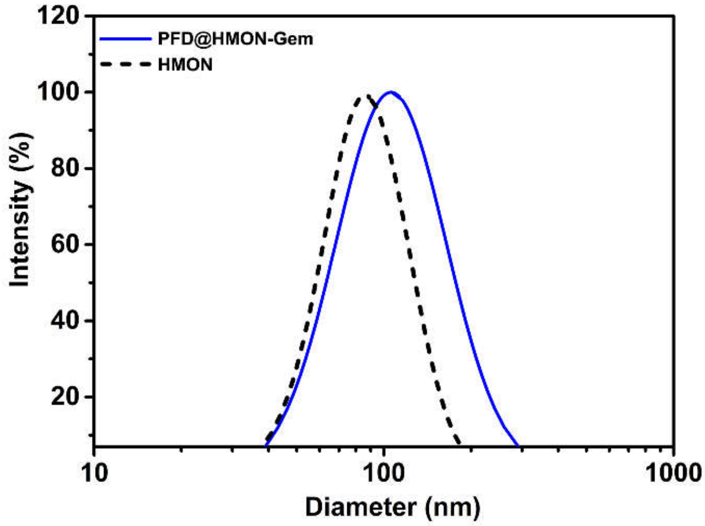

Figure S5. Hydrodynamic diameters of the HMON and PFD@HMON-Gem.

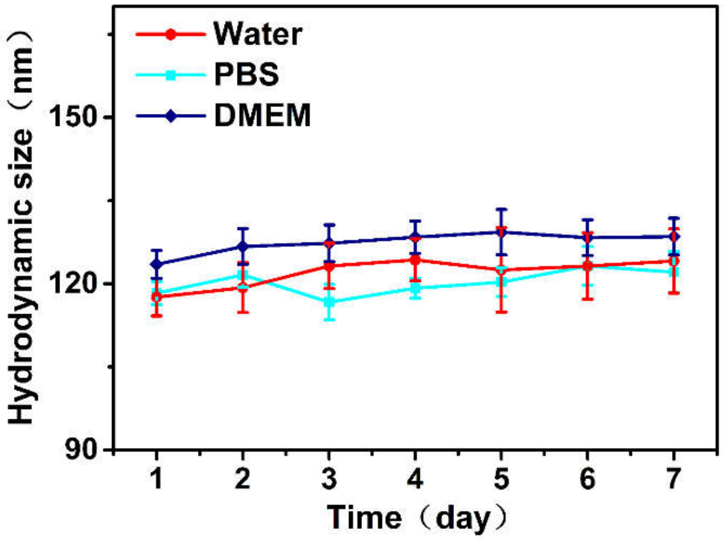

Figure S6. The stability of PFD@HMON-Gem nanoparticles in water, PBS or DMEM medium was investigated by measuring the hydrodynamic size of the particles.

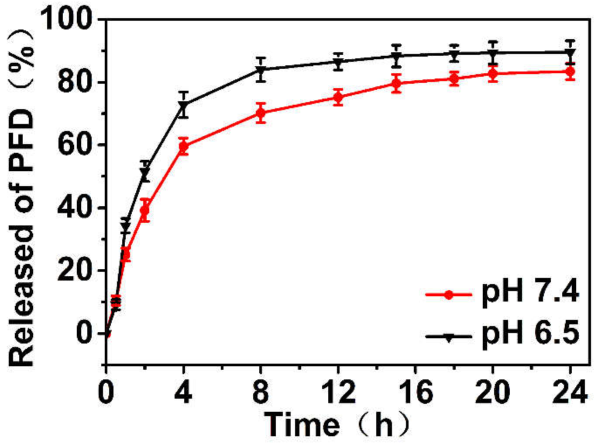

Figure S7. *In vitro* PFD release profiles from PFD@HMON at pH 7.4 or 6.5 in PBS.

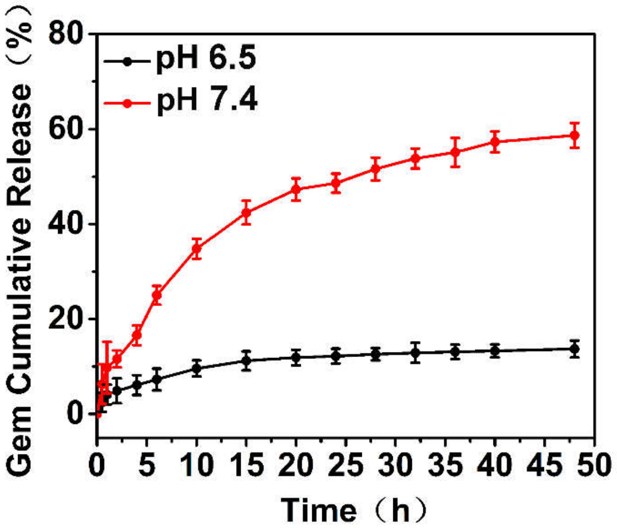

Figure S8. *In vitro* gemcitabine release profiles from PFD@HMON-Gem at different pH values.

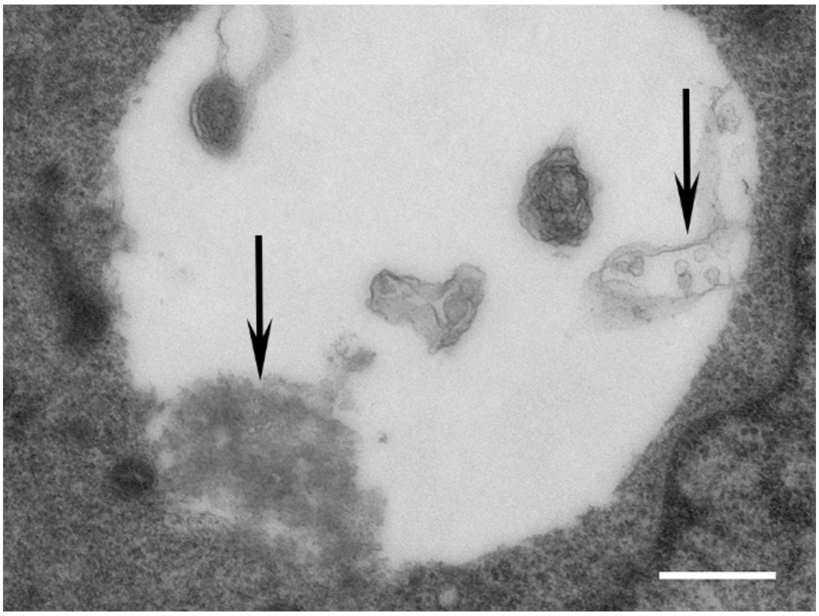

Figure S9. Intracellular biodegradation assay by direct bio-TEM observation. The arrows indicate the structural collapse and nanoparticle dissolution of HMON. (bar = 500nm)

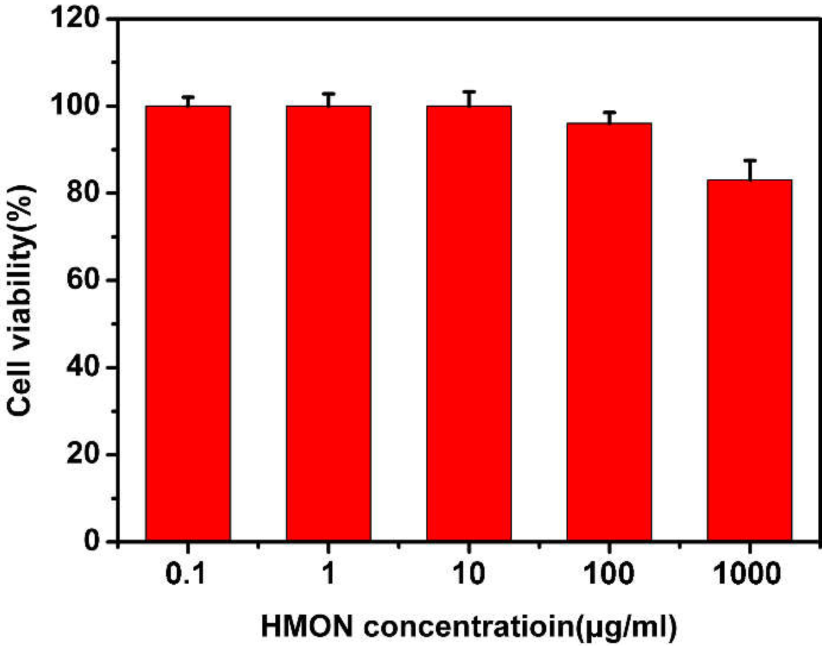

Figure S10. Cell viability of HUVEC cells after incubated with HMON for 24 h.

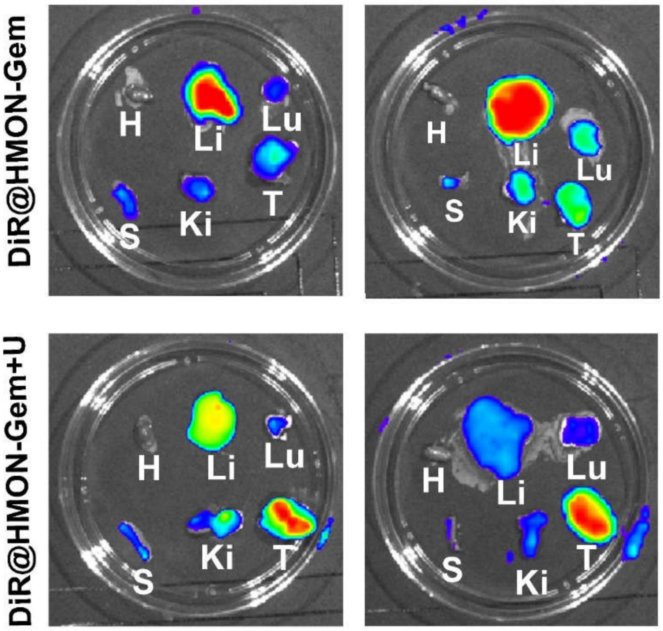

Figure S11. *Ex vivo* DiR fluorescence images of tumors and major organs (Li: liver, S: spleen, Ki: kidney, Lu: lung, H: heart and T: tumor).

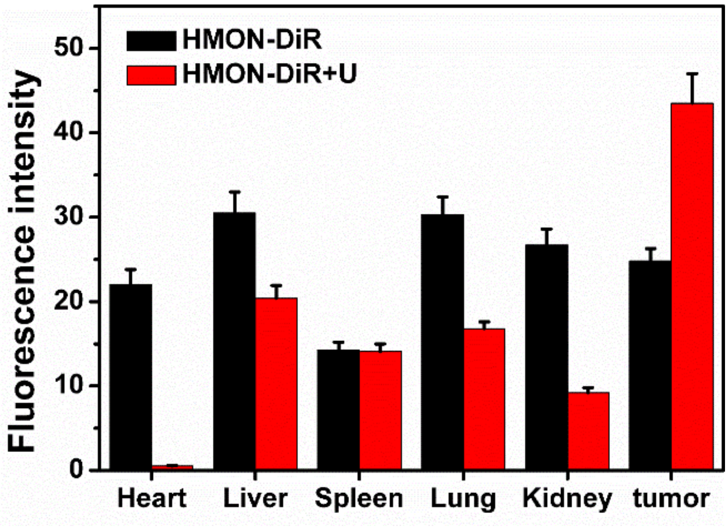

Figure S12. Statistical analysis of hearts, livers, spleens, lungs, kidneys and tumors harvested after sacrifice (n = 3, results are shown as mean ± S.D.).

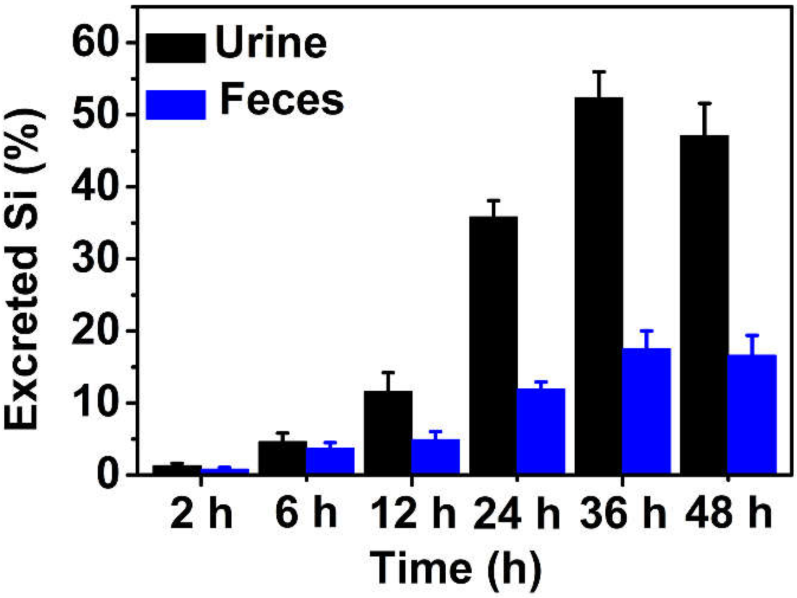

Figure S13. Time-dependent distribution by measuring Ir concentration of PFD@HMON-Gem NPs in urine and feces collected at various time points after injection.

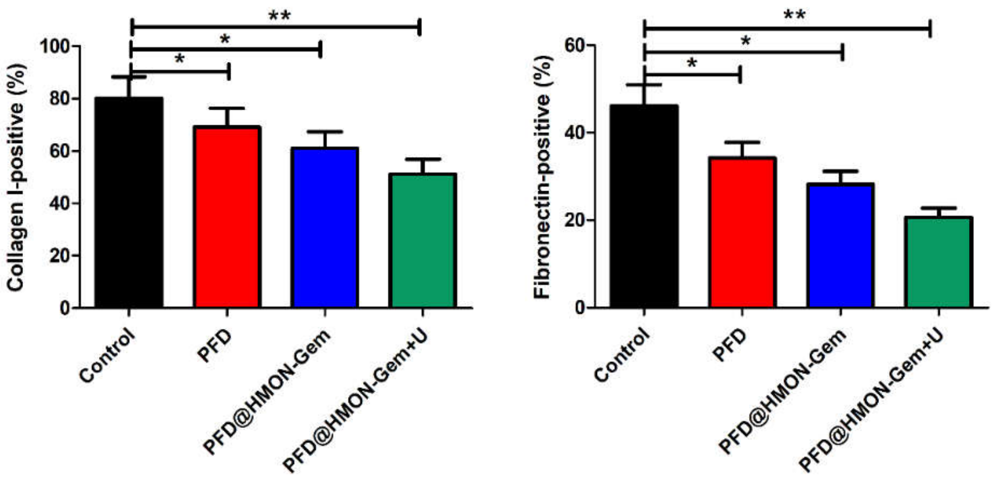

Figure S14. Quantitative analysis of collagen I/fibronectin-positive staining areas in each group (mean ± S.D., $n = 5$), * $P < 0.05$, ** $P < 0.01$ as compared to control group.

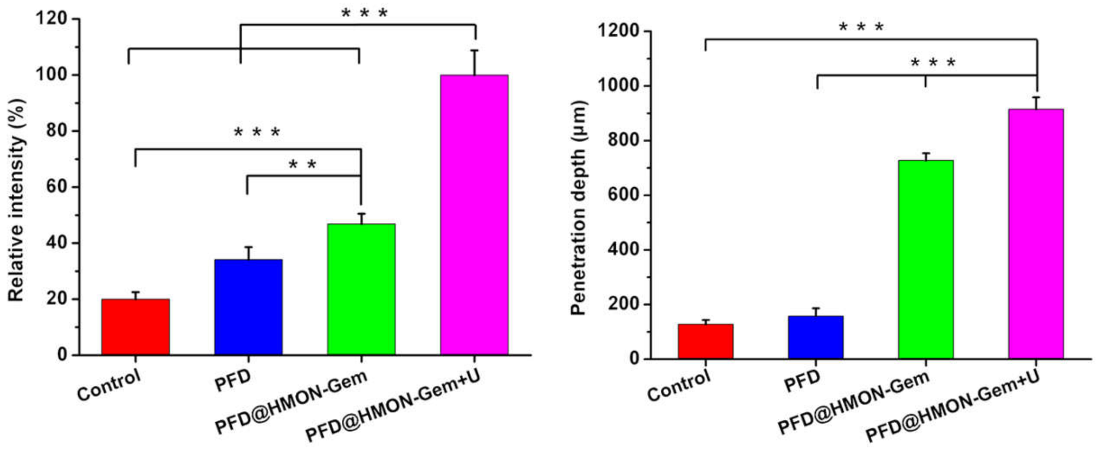

Figure S15. Rhodamine relative intensity and penetration depth in tumor.

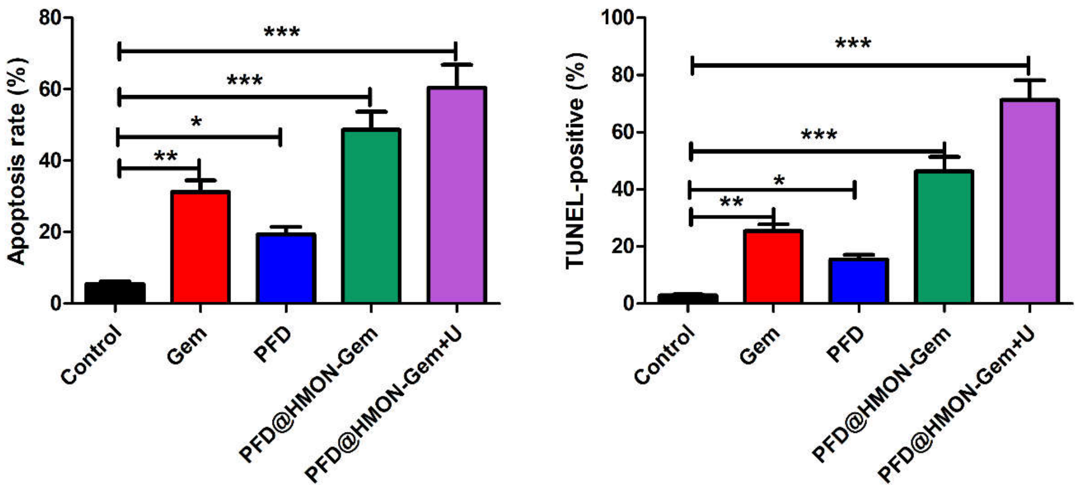

Figure S16. Quantitative analysis of cell apoptosis and TUNEL-positive in each group (mean ± S.D., n = 6), * $P < 0.05$, ** $P < 0.01$ and *** $P < 0.001$ as compared to control group.

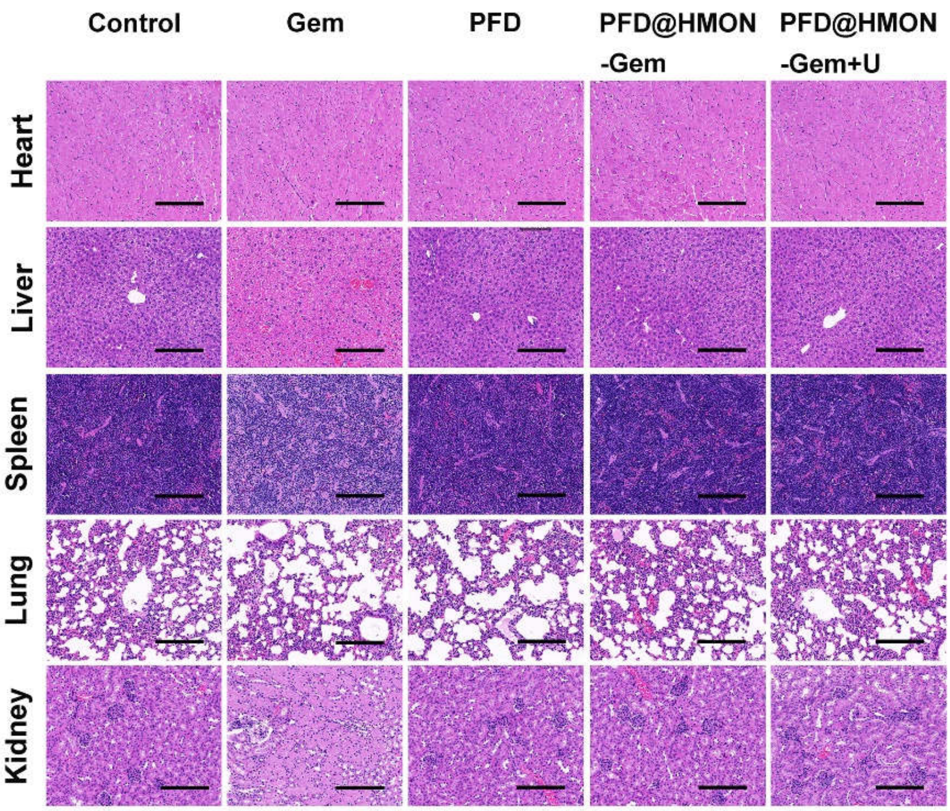

Figure S17. Representative H&E sections of organ tissues (heart, liver, spleen, lung, and kidney) of tumor-bearing mice after treatment with either saline, Gem, PFD, PFD@HMON-Gem or PFD@HMON-Gem + UTMD. The scale bar, 200 μm.

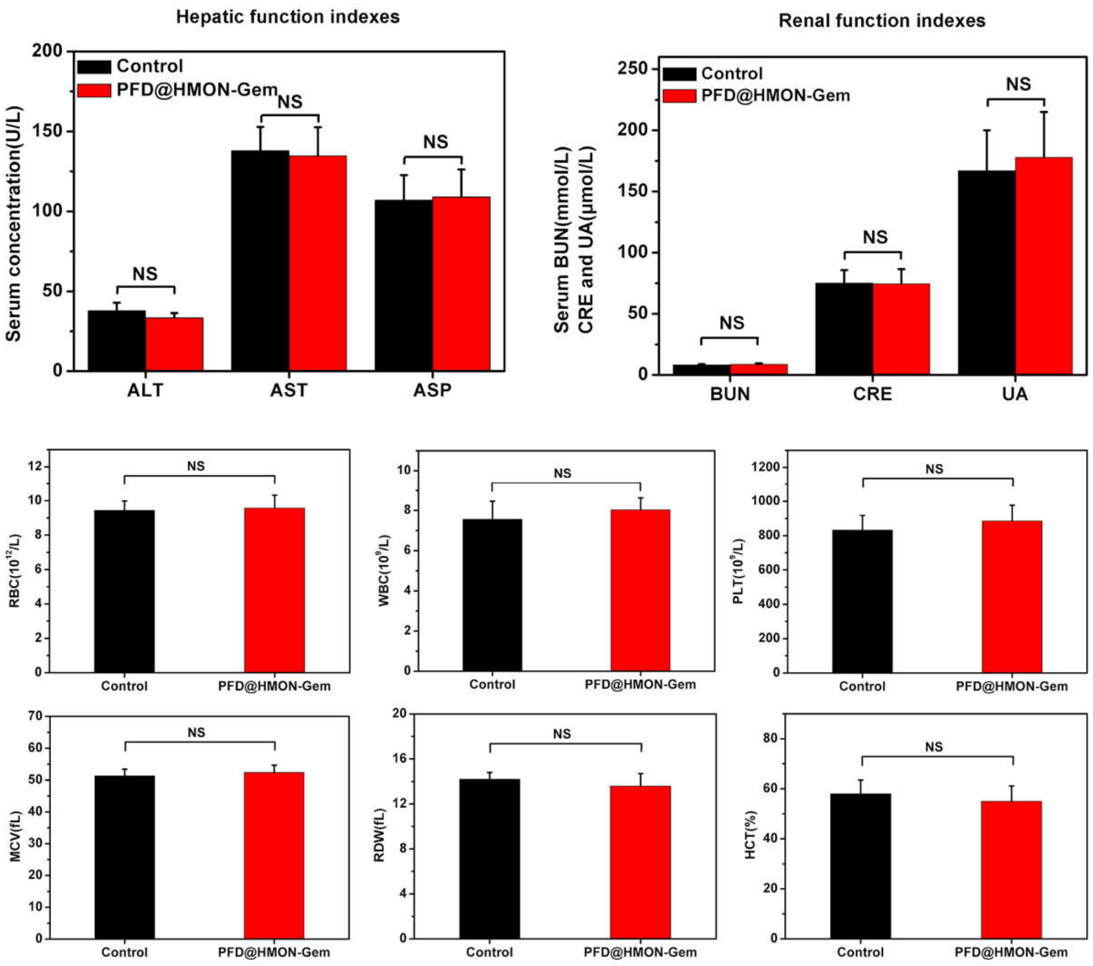

Figure S18. The blood cell analysis and the detection of routine biochemistry indicators.

Table S1. The values for describing Figure 3E.

Time (h)	0	3	6	12	18	24	36	72	96	120	144	168	240	360
**Diameter (nm)**	**87.7**	**79**	**76.8**	**68**	**54**	**46**	**41.8**	**39.3**	**38.4**	**32.1**	**30.3**	**29.8**	**25.2**	**21**
